# LTP or LTD? Modeling the Influence of Stress on Synaptic Plasticity

**DOI:** 10.1523/ENEURO.0242-17.2018

**Published:** 2018-04-06

**Authors:** Achim Peters, Cordula Reisch, Dirk Langemann

**Affiliations:** 1 Medical Clinic 1, Endocrinology and Diabetes, University of Luebeck, Luebeck D-23538, Germany; 2 Institut Computational Mathematics, Technical University Braunschweig, Braunschweig D-38106, Germany

**Keywords:** cognitive memory, glucocorticoids, long-term depression, long-term potentiation, mathematical model, stress

## Abstract

In cognitive memory, long-term potentiation (LTP) has been shown to occur when presynaptic and postsynaptic activities are highly correlated and glucocorticoid concentrations are in an optimal (i.e., low normal) range. In all other conditions, LTP is attenuated or even long-term depression (LTD) occurs. In this paper, we focus on NMDA receptor (NMDA-R)-dependent LTP and LTD, two processes involving various molecular mechanisms. To understand which of these mechanisms are indispensable for explaining the experimental evidence reported in the literature, we here propose a parsimonious model of NMDA-R-dependent synaptic plasticity. Central to this model are two processes. First, AMPA receptor-subunit trafficking; and second, glucocorticoid-dependent modifications of the brain-derived neurotrophic factor (BDNF)-receptor system. In 2008, we have published a core model, which contained the first process, while in the current paper we present an extended model, which also includes the second process. Using the extended model, we could show that stress attenuates LTP, while it enhances LTD. These simulation results are in agreement with experimental findings from other labs. In 2013, surprising experimental evidence showed that the GluA1 C-tail is unnecessary for LTP. When using our core model in its original form, our simulations already predicted that there would be no requirement for the GluA1 C-tail for LTP, allowing to eliminate a redundant mechanism from our model. In summary, we present a mathematical model that displays reduced complexity and is useful for explaining when and how LTP or LTD occurs at synapses during cognitive memory formation.

## Significance Statement

Synaptic plasticity is a complex process. For the description of biological processes, the best mathematical model is characterized by low complexity and high accuracy. Here, we present a “low-complexity” model of synaptic plasticity that predicts the available experimental data with “high accuracy.” Among other things, one can use the model to explain how stress affects the occurrence of NMDA-R-dependent LTP and LTD.

## Introduction

For decades, stress has been reported to affect memory formation (de Kloet et al., 1998; Joëls, 2006; Popoli et al., 2011). Stress-induced rises in glucocorticoid concentrations have been shown to exert differential effects on cognitive and emotional memories (Maggio and Segal, 2012). While stress typically impairs cognitive memories, it may enhance emotional memories as is the case of fear memory (Quirarte et al., 1997). The current paper focuses on the effect of glucocorticoids on cognitive memories. Particularly, we will focus on NMDA receptor (NMDA-R)-dependent synaptic potentiation (LTP) and depression (LTD), two forms of activity-dependent long-term changes in synaptic efficacy that have been extensively studied in brain slice cultures. Glucocorticoids affect NMDA-dependent synaptic plasticity, which typically occurs in the dorsal hippocampus. Here as well as in other brain regions, glucocorticoids affect intracellular mineralocorticoid receptors (MRs) and glucocorticoid receptors (GRs). MRs show a high affinity to glucocorticoids, while GRs show low affinity (Arriza et al., 1988). There is experimental evidence that in the dorsal hippocampus stress reduces long-term potentiation (LTP), while it enhances long-term depression (LTD; Maggio and Segal, 2007, 2009).

A key link between glucocorticoid actions and LTP probability is the signaling of brain-derived neurotrophic factor (BDNF): particularly the production of its high-affinity receptors TrkB and its low-affinity receptors p75. TrkB receptor signaling facilitates LTP (Minichiello et al., 1999; Zhang and Poo, 2002), while p75 receptor signaling facilitates LTD (Rösch et al., 2005; Woo et al., 2005). Glucocorticoid-activated MR and GR receptors control the production rates of TrkB and p75 receptors. A series of elegant experiments manipulating glucocorticoid concentrations has demonstrated that at low glucocorticoid concentrations MRs enhance TrkB-production and LTP (Stranahan et al., 2008, 2010, 2011), while at high glucocorticoid concentrations GRs suppress TrkB-production and LTP (Wosiski-Kuhn et al., 2014). In this way, TrkB production is maximal at low (normal) glucocorticoid concentration and minimal in the absence of glucocorticoids or at high glucocorticoids concentration.

In addition to stress, a second factor also determines the induction and maintenance of LTP and LTD. This is correlated activity between the presynaptic and postsynaptic neuron. For LTP induction both pre- and postsynaptic neurons need to be active at the same time because the postsynaptic neuron must be depolarized when glutamate is released from the presynaptic bouton to fully relieve the Mg^++^ block of NMDA-Rs. There is extensive evidence that modest activation of NMDA-Rs leading to modest increases in postsynaptic calcium is optimal for triggering LTD, while a much stronger activation of NMDA-Rs leading to much greater increases in postsynaptic calcium, is required to trigger LTP (Luscher and Malenka, 2012; Miyashita et al., 2012). Since the direction of synaptic plasticity depends on the timing between the presynaptic and postsynaptic spikes, this phenomenon has been called “spike-timing-dependent plasticity” (Bi and Poo, 2001). If a presynaptic spike is repetitively elicited slightly before the postsynaptic neuron is fired, the excitatory postsynaptic potential precedes the backpropagating action potential, and such repetitive “pre-post” action potential firing can generate LTP. Conversely, when the backpropagating action potential is repetitively elicited before the presynaptic spike, “post-pre” firing, LTD is often observed. Thus, NMDA-Rs, and in some cases the voltage-dependent calcium channels (VDCCs), may function as coincidence detectors (Luscher and Malenka, 2012; Miyashita et al., 2012).

Both factors, the effects of glucocorticoids (indicating the presence of stress) and the effects of NMDA-Rs (indicating correlated pre-/postsynaptic activity) converge in one common point. This is the interaction between the ligand BDNF and its TrkB receptor. If there is coincidental activity of the pre- and postsynaptic neuron, proBDNF is converted to mature BDNF (mBDNF), and in this way the concentrations of mBDNF in the synaptic cleft rise. If glucocorticoid concentrations are low (normal), the number of postsynaptically expressed TrkB receptors is maximal. Thus, if many BDNF molecules can interact with a large number of TrkB receptors, LTP is promoted.

**Figure 1.**
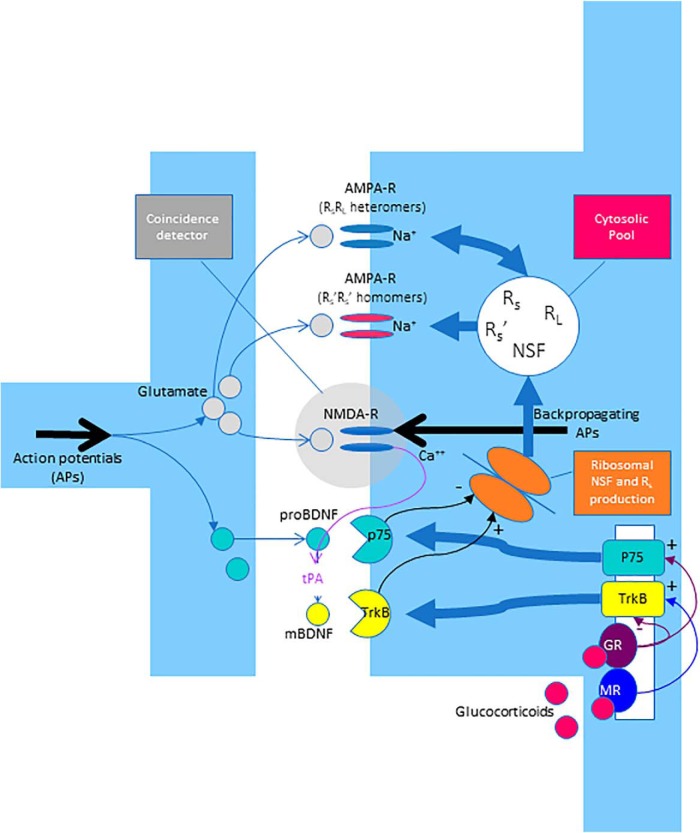
Modeling the glucocorticoid dependency of NMDA-R-dependent LTP and LTD. The core model, published earlier (Langemann et al., 2008), describes the trafficking of AMPA receptor subunits. In brief, a cytosolic pool consists of short-tailed AMPA receptor subunits (R_S_), long-tailed AMPA receptor subunits (R_L_), and NSF. R_S_ consist of the short-tailed AMPA receptor subunits GluA2 or GluA3; R_L_ consist of GluA1 or GluA4. In case of high numbers of R_S_ and NSF molecules, the probability of NSF binding to R_S_ is high, and in this way, the formation of activated R’_S_ is promoted. Activated R’_S_ form R’_S_R’_S_ homomers, which in turn endorse further activation of existing R_S_ into R’_S_. In case of low numbers of R_S_ and NSF molecules, nonactivated R_S_ prevail. Nonactivated R_S_ promote the formation of R_S_R_L_ heteromers, which in turn endorse deactivation of existing R’_S_ into R_S_. Thus, there is a double positive feedback loop. First, the occurrence of AMPA receptor homomers increases numbers of R’_S_, which in turn endorses the formation of more AMPA receptor homomers. Second, the occurrence of AMPA receptor heteromers increases the number of R_S_, which in turn endorses the formation of more AMPA receptor heteromers. The extended model, as presented in the current article, describes how correlated pre/postsynaptic activity and glucocorticoid concentrations modify ribosomal R_S_ and NSF production. Incoming action potentials elicit glutamate release. Glutamate can only activate NMDA-Rs if the postsynaptic cell is coincidentally depolarized by backpropagating action potentials. Therefore, the NMDA-R functions as a coincidence detector. On NMDA-R activation, tPA is released into the synaptic cleft, thereby converting proBDNF into mBDNF. Hence, high concentrations of mBDNF indicate highly correlated activity between the pre- and the postsynaptic neuron. mBDNF primarily binds to TrkB receptors, while proBDNF primarily binds to p75 receptors. The numbers of TrkB and p75 receptors expressed on the postsynaptic density depend on glucocorticoid concentrations. Glucocorticoids bind with high affinity to MRs and with low affinity to GRs. Low (normal) glucocorticoid concentrations promote (via MRs) the production of TrkB receptors, while high glucocorticoid concentrations (via GRs) inhibit TrkB production and favor p75 production. Hence, a large number of TrkB receptors on the postsynaptic density indicates low (normal) glucocorticoid concentrations. On interaction between mBDNF and its TrkB receptors, ribosomal NSF and R_S_ production rates are enhanced; on interaction between proBDNF and its p75 receptors, ribosomal NSF and R_S_ production rates are suppressed. Consequently, the interaction between correlated pre/postsynaptic activity and glucocorticoid concentrations determine the proportional distribution of R_S_, R_L_, and NSF in the cytosolic pool. In turn, the proportional distribution of R_S_, R_L_, and NSF in the cytosolic pool determines the number of AMPA receptor homomers in the postsynaptic density: an increased number of AMPA receptor homomers indicates LTP, a decreased number of AMPA receptor homomers indicates LTD.

In the current paper, we present a mathematical model that uses differential equations for describing how stress affects the BDNF/TrkB-receptor system, and how the BDNF/TrkB-receptor system influences AMPA receptor trafficking (Fig. 1). Our model is based on a “core model” on AMPA receptor trafficking that we had published earlier (Langemann et al., 2008). Here, we use an “extended model” which adds the stress-dependent processes that modify the BDNF/TrkB-receptor system. Using experimental findings from another lab (Maggio and Segal, 2007, 2009), we show that our extended model allows to predict how stress influences the induction and maintenance of LTP or LTD in cognitive memory formation.

## Materials and Methods

The plasticity model discussed in (Langemann et al., 2008) is shortly presented and extended by a modeling step regarding the stress level and the coincidence of pre- and postsynaptic activity. The new modeling step describes mechanisms which lie causally before the core plasticity model.

### Code accessibility

The code is accessible as Extended data.

10.1523/ENEURO.0242-17.2018.ed1 Extended Data 1 The code is composed of a main program (1st part) and three subprograms (2nd to 4th part). The program can be executed by using MATLAB software. Download Extended Data 1, ZIP file.

### Biochemical reaction kinetics

Synaptic plasticity depends on the amount of activated (R’_S_) and nonactivated (R_S_) short tail AMPA subunits (Shi et al., 2001). NEM-sensitive factor (NSF) activates short-tail AMPA subunits (Nishimune et al., 1998). This reaction can be written as

$$R_{S}+NSF\overset{k_{0},k_{4}}{↔}{R\prime }_{S},$$

where *k_0_* and *k_4_* are nonlinear reaction parameters. Therein, *k_0_* depends on the amount of activated R’_s_R’_s_ homomers, and *k_4_* is influenced by nonactivated R_s_R_L_ heteromers. The polymerizations, leading to homomeric and heteromeric AMPA units (Esteban, 2003), are

$$2{R\prime }_{S}\overset{k_{1},k_{2}}{↔}{R\prime }_{S}{R^{\prime }}_{S},$$

and

$$R_{S}+R_{L}\overset{k_{5},k_{6}}{↔}R_{S}R_{L}$$

with constant reaction rates *k_1_*, *k_2_*, *k_5_*, and *k_6_* for both directions of the bidirectional polymerization reactions. As mentioned in (Langemann et al., 2008), not only dimers but also tetramers take part in the reactions. The occurrence of tetramers depends on the concentration of activated short-tail units. Therefore, the production of tetramers can be seen as a secondary reaction. We regard the occurrence of dimeric polymers as qualitative representative for the presence of realistic tetrameric homomers and heteromers.

**Figure 2.**
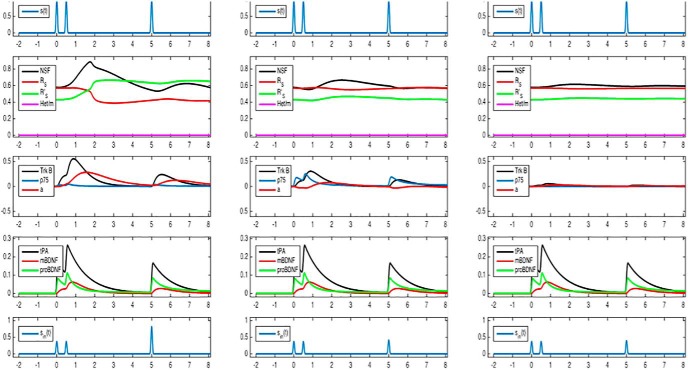
Nonlinear kinetics. Left, k_0_ depends on activated short-tailed AMPA receptor subunits (R’_S_). Right, k_4_ depends on nonactivated short-tailed AMPA receptor subunits (R_S_).

The polymerization influences the activation of R_S_ (Langemann et al., 2008). To be more precise, the activation reaction parameter *k_0_* depends nonlinearly on the concentration of activated homomers R’_S_R’_S_ and the deactivation reaction parameter *k_4_* depends nonlinearly on the concentration of nonactivated heteromers R_S_R_L_. The activated homomeric and nonactivated heteromeric dimers depend on activated or, respectively, nonactivated short-tailed subunits. As a result, we get nonlinear dependencies *k_0_* ([R’_S_]) and *k_4_* ([R_S_]), and very simple examples of them are shown in Figure 2.

Altogether, we get reaction kinetics for the main components R_S_, R’_S_, and NSF by

$$\left[N\dot{S}F\right]=p_{NSF}-z_{NSF}[NSF]-k_{0}({R\prime }_{S})\cdot \left[R_{S}\right][NSF]+k_{4}(R_{S})\cdot \left[{R\prime }_{S}\right],$$

$$[{\dot{R}}_{S}]=p_{R_{S}}-z_{R_{S}}\left[R_{S}\right]-k_{0}({R\prime }_{S})\cdot \left[R_{S}\right][NSF]+k_{4}(R_{S})\cdot \left[{R\prime }_{S}\right],$$

$$[{{\dot{R}}^{\prime }}_{S}]=-z_{{R\prime }_{S}}\left[{R\prime }_{S}\right]+k_{0}({R\prime }_{S})\cdot \left[R_{S}\right][NSF]-k_{4}(R_{S})\cdot \left[{R\prime }_{S}\right],$$

where *p*_#_ are production rates and *z*_#_ are decay parameters of substance #.

The ribosomal protein production rates of NSF and R_s_ are increased by TrkB receptors (Minichiello et al., 1999; Narisawa-Saito et al., 1999; Zhang and Poo, 2002) and decreased by p75 (Rösch et al., 2005; Woo et al., 2005). These numbers of membrane TrkB and p75 receptors depend on cortisol concentrations and thus on stress. This is because glucocorticoid-activated MR and GR receptors control the production rates of TrkB and p75 receptors. In detail, MR enhances TrkB receptor production (Schaaf et al., 1997); GR counteracts TrkB receptor production (Schaaf et al., 1997) and promotes the production of p75 receptors (Shi and Mocchetti, 2000).

We distinguish two main factors for inducing and maintaining LTP or LTD: first, correlated/noncorrelated pre-/postsynaptic activity and, second, the presence/absence of stress. The first concerns the coincidence of the incoming action potentials, i.e., the signal *s(t)*, with backpropagating action potentials. The incoming signal causes a production of activated calmodulin-dependent protein kinase II (CaMKII) ([CaMKII]) as in

$$[Ca\dot{M}KII]=b_{CaMKII}s(t)-z_{CaMKII}[CaMKII],$$

where *b*_CaMKII_ describes the influence of glutamate on the production of CaMKII. Besides, glutamate triggers a release of tissue plasminogen activator ([tPA]) into the synaptic cleft as in

$$[t\dot{P}A]=\sigma s(t)-z_{tPA}[tPA],$$

where σ is a parameter for the coincidence of pre/postsynaptic activity (pre: glutamate release/post: backpropagating action potentials; Gualandris et al., 1996). The NMDA-R serves as a coincidence detector, as it allows Ca^++^ influx only during correlated activation of both pre- and postsynaptic cells (Miyashita et al., 2012). If there is highly correlated pre/postsynaptic activity, σ is large; otherwise, σ is small or zero. tPA influences BDNF. In detail, tPA, by activating the extracellular protease plasmin, converts the precursor proBDNF to the mBDNF (Pang et al., 2004). The reaction can be described as

$$proBDNF+tPA\overset{k_{8},k_{9}}{↔}mBDNF,$$

where *k_8_* is a parameter for the forming of mBDNF and *k_9_* is a parameter for the forming proBDNF.

Stress increases the cortisol concentrations [C]. Cortisol binds to intracellular MRs with high affinity and to GRs with low affinity (Arriza et al., 1988). We describe these reactions with Michaelis–Menten kinetics

$$[MR]=\frac{\left[C\right]}{[C]+M_{MR}},\;[GR]=\frac{\left[C\right]}{[C]+M_{GR}},$$

where *M*_MR_ and *M*_GR_ are Michaelis constants for saturated production of MR and GR, respectively. The preferred binding of cortisol with MR is expressed by *M*_MR_ ≪ *M*_GR_.

Activated MR and GR regulate the expression of TrkB and p75 receptors. MR has a positive effect on TrkB expression; GR has a positive effect on the expression of p75 and a negative effect on TrkB (Schaaf et al., 1997; Shi and Mocchetti, 2000). Hence, the amount of TrkB and p75 receptors depend on the stress level. ProBDNF binds to p75 with a high affinity (Lee et al., 2001). In contrast, mBDNF binds to TrkB with high affinity and to p75 with a low affinity (Huang and Reichardt, 2003).

Activated proBDNF [*pBDNF_act_*] and mBDNF [*mBDNF_act_*] influence the production rates of NSF and R_S_. The production rate increases with active mBDNF (Minichiello et al., 1999; Narisawa-Saito et al., 1999; Zhang and Poo, 2002) and decreases with proBDNF (Rösch et al., 2005; Woo et al., 2005) like

$$\dot{a}=b_{a}\left(\left[pBDNF_{act}\right]-\left[mBDNF_{act}\right]\right)-z_{a}a,$$

where *b_a_* is a parameter for the influence of the neurotrophic factor and *z*_a_ is the linear decay rate. This is the point, where the new mechanisms docks onto the core model of (Langemann et al., 2008).

In the same way, as in Langemann et al. (2008), we describe the signal transmission dependent on the homomeric and heteromeric AMPA units in the membrane. Therefore, we use catalytic reactions for the transfer of R_S_R_L_ in the membrane

$$R_{S}R_{L}+CaMKII\overset{k_{11}}{\to }R_{S}R_{L}{}^{mem}+CaMKII,$$

and for the opposed reaction with decomposition

$$R_{S}R_{L}{}^{mem}+Glut\overset{k_{12}}{\to }R_{S}+R_{L}+Glut.$$

Furthermore, heteromeric units vanish from the membrane

$$R_{S}R_{L}{}^{mem}\overset{k_{12}}{\to }R_{S}R_{L}.$$

The amount of activated homomeric AMPA units in the membrane is proportional to the amount of activated homomeric units,

$$\left[{R\prime }_{S}{R\prime }_{S}\right]\propto \left[{R\prime }_{S}{R\prime }_{S}{}^{mem}\right].$$

Based on these reaction, the transmitted signal is modeled by

$$s_{in}(t)=2\left(\left[{R\prime }_{S}{R\prime }_{S}{}^{mem}\right]+\left[R_{S}R_{L}{}^{mem}\right]\right)s(t).$$

### Parameter choice

Besides the time scale, which is realistic, the concentrations are normalized. Except from a few parameters, the parameters are normalized to 1. Exceptions are the Michalis constants *M*_MR_ = 0.0001 and *M*_GR_ = 5. The sensitivity of the production rate of NSF depending on TrkB is *u*_NSF_ = 3 and therefore higher than the other sensitivities. The decay of tPA and proBDNF is slower than the other reactions, z_tPA_ = 0.5 and z_proBDNF_ = 0.1. In contrast, the decay of active TrkB and active p75 are faster, z_actTrkB_ = 2 and z_actp75_=2. BDNF, active TrkB and tPA have a higher production rate with p_BDNF_ = 10, p_actTrkB_ = 10 and p_tPA_ = 2. The used parameter set is simple enough, and the qualitative behavior of the system of differential equations do not depend on a single chosen parameter or its variation.

### Model properties

As discussed in Langemann et al. (2008), the rates of change $\left[\dot{NSF}\right]$, $\left[\dot{R_{s}}\right]$, and $\left[\dot{{R'}_{s}}\right]$ are closely related to each other because the interaction terms with the reaction rates *k_0_* ([R_s_’]) and *k_4_* ([R_s_]) occur in all three ordinary differential equations describing the reaction kinetics. Therefore, these three differential equations can be reduced to a single differential equation, e.g., in the concentration [R’_s_]. Now, the properties of the dynamical system can be discussed by hands of the production and decay terms of R’_s_.

**Figure 3.**
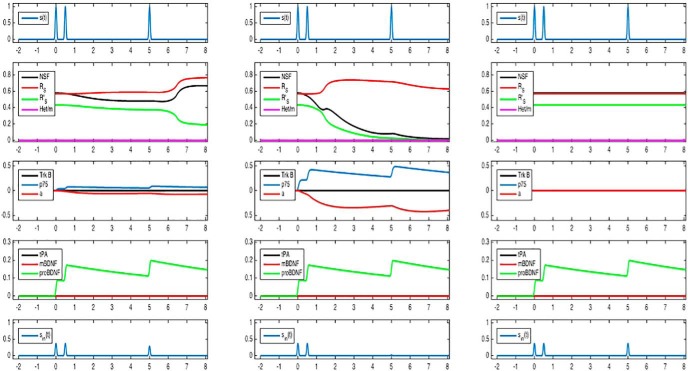
The number of stable stationary points depends on the NSF production rate. Left, With normal NSF production rates (p_NSF_), we find five stationary points, three stable stationary points (active, LTD, and LTP) separated by two unstable points. Middle, A low NSF production rate (p_NSF_) shifts the production and the decay term of R’_s_ so that only the stable stationary points remains which belongs to LTD. Right, A high NSF production rate (p_NSF_) drives the system into the only other stable stationary point which belongs to LTP.

The kinetics *k_0_* ([R_s_’]) and *k_4_* ([R_s_]) resulting from the polymerization of R’_s_ and R_s_, respectively, have an S-shape and a self-excitation of each R'_S_ and of R_s_. Both self-excitations are in competition to each other. Exemplary kinetics are given in Figure 2. The kinks are chosen for a better visibility of the interaction behavior in Figure 3. The qualitative behavior is conserved for more realistic smooth S-shaped kinetics. Under standard levels of p_NSF_, we find five stationary points, three stable stationary points separated by two unstable points (Figure 3, left panel). The stable stationary point with a low concentration of R’_s_ is associated with LTD, and the stable stationary point with a high concentration of R’_s_ is associated with LTP because the concentration of R’_s_ is closely related to the occurrence of AMPA homomers in the membrane. The stable stationary points stand for an active synapse which is not yet in LTD or LTP.

A more detailed analysis of the system of reaction kinetics works as follows: Stationary points are characterized by vanishing time derivatives of [NSF], [R_S_], and [R’_S_]. As mentioned in (Langemann et al., 2008), all three kinetic equations contain the same interaction term between R_S_ and NSF. Hence, in the stationary case, we get the complementary relation

$$p_{NSF}-z_{NSF}\left[NSF\right]=p_{R_{s}}-z_{R_{s}}\left[R_{s}\right]=z_{R_{s}^{'}}\left[R_{s}^{'}\right]$$

leading to the relations

$$\left[R_{s}\right]=\frac{p_{R_{s}}-z_{R_{s}^{'}}\left[R_{s}^{'}\right]}{z_{R_{s}}}\text{ and }\left[NSF\right]=\frac{p_{NSF}-z_{R_{s}^{'}}\left[R_{s}^{'}\right]}{z_{NSF}}$$

in every stationary state of the synaptic system. We insert these relations in the last of the three kinetic equations, again for the stationary case, and we find

$$0=\underset{\text{D}}{\underset{︸}{\underset{\;}{-\left(z_{{R\prime }_{S}}+k_{4}\right)[{R\prime }_{S}]}}}+\underset{\text{P}}{\underset{\;}{\underset{︸}{k_{0}\frac{p_{R_{S}}-z_{{R\prime }_{S}}[{R\prime }_{S}]}{z_{R_{S}}}\cdot \frac{p_{NSF}-z_{{R\prime }_{S}}[{R\prime }_{S}]}{z_{NSF}}}}}$$

which is an algebraic relation in only one variable, namely in [R’_S_]. The decay term D, which is always negative, and the positive production term P are equilibrated in every stationary point. They are shown in Figure 3 as blades of the scissors.

A standard concentration of NSF (Fig. 3, middle panel) leads to a middle intensity of the production term, and due to the S-shape of the self-activation curve, we get five stationary points. Three of them are stable, and they are activated with the LTD equilibrium, the active middle state and the LTP equilibrium. A transition of the system from one state into another is not immediately reversed and therefore, it means synaptic plasticity. Such a transition is mediated by a high or low NSF concentration over a time interval. A high production of NSF increases the blade standing for the production term, and only LTP persists as stable equilibrium (Fig. 3, right panel). Therefore, a temporarily increase of the NSF-production moves the system into LTP. Analogously, a temporarily decrease of the NSF-production decreases the production term P, and only the LTD equilibrium survives.

A temporarily decreased level of p_NSF_ shifts the production and the decay term of R’_s_ so that only the stable stationary point remains, which belongs to LTD, and the relatively fast reaction system tends to this equilibrium (Fig. 3, middle panel). After a consequent re-increase of the p_NSF_-level, the system slowly moves into the LTD equilibrium. Vice versa, a temporarily increased level of p_NSF_ drives the system into LTP (Fig. 3, right panel). In this way, the plasticity behavior of a synapse can be traced back to reaction kinetics of simple biochemical reactions without any memory tag or unknown mechanism. Here, this core model is extended by the influence of stress hormones and by the impact of signal coincidence as discussed in the following paragraph.

### Model hierarchy

The presented extension of the model from Langemann et al. (2008) concerns the influence of stress and the coincidence of the incoming signals on LTP or LTD, respectively, and lets the core model of the synaptic plasticity unchanged. The core model and the extension can be regarded as two steps of modeling with sequential interaction.

The first modeling step transforms the input quantities stress, described by the cortisol rate, and the coincidence of the signals, into the output rates of MR, GR, proBDNF, and mBDNF and thus the amount of active TrkB, p75, NSF, and R_S_. Their concentrations enter the second modeling step, which is just the original core model.

On the one hand, the entire model presented here is a model extension, which allows to discuss the influence of stress and the coincidence of the incoming signals. On the other hand, the original core model is an independent sub-model in the entire model, and the entire model inherits the properties of the core model. This is the reason why, we refer for all discussion of the plasticity behavior itself to (Langemann et al., 2008).

## Results

**Figure 4.**
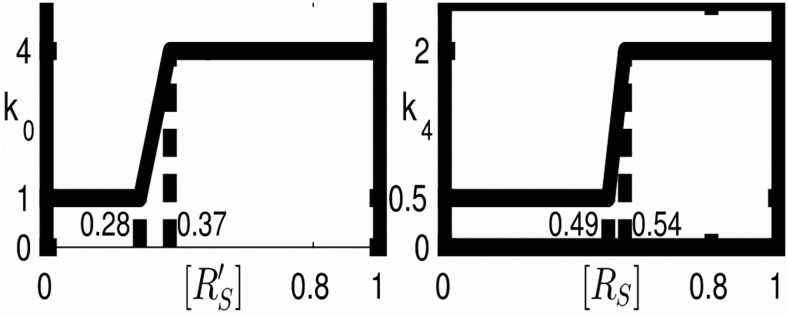
Simulation of correlated pre-/postsynaptic activity at three levels of cortisol concentrations. ***A***, Low cortisol. With correlated activity, mBDNF concentrations rise (4th panel, red curve). At low cortisol concentrations, the number of TrkB receptors is high. When high mBDNF concentrations meet a high number of TrkB receptors, TrkB signaling is enhanced (3rd panel, black curve). Consequently, protein production (R_s_ and NSF) increases (3rd panel, red curve). As a result, the probability that NSF will bind to R_s_ increases, so that the activated form R_s_’ will prevail (2nd panel, the green curve becomes higher than the red curve). Thus, these simulations show that low cortisol concentrations combined with correlated pre-/postsynaptic activity produce a full-magnitude LTP of approximately double size as compared to baseline (5th panel; increased excitatory postsynaptic potentials at time 5, interpreted as hours). Comparison with experimental data, In nonstressed animals, high-frequency stimulation enhanced LTP by a factor of 1.69 compared to baseline (Maggio and Segal, 2007). ***B***, High cortisol. With correlated activity, mBDNF concentrations rise (4th panel, red curve). At high cortisol concentrations, the number of TrkB receptors is low and the number of p75 receptors is high. When high mBDNF concentrations meet a small number of TrkB receptors, TrkB signaling is weak (3rd panel, black curve). Consequently, protein production (R_s_ and NSF) is only modestly increased (3rd panel, red curve). As a result, the probability that NSF will bind to R_s_ is only modestly increased, so that the nonactivated form R_s_ will prevail (2nd panel, the green curve remains lower than the red curve). Thus, these simulations show that high cortisol concentrations combined with correlated pre-/postsynaptic activity enhanced LTP only slightly (5th panel at time 5; compare Fig. 1). Comparison with experimental data, In stressed animals, high-frequency stimulation enhanced LTP by a factor of 1.42 only (Maggio and Segal, 2007). ***C***, No cortisol. With correlated activity, mBDNF concentrations rise (4th panel, red curve). However, without cortisol, the number of TrkB receptors is low, and therefore TrkB signaling is almost absent (3rd panel, black curve). Consequently, protein production (R_s_ and NSF) is unaltered (3rd panel, red curve). Thus, these simulations show that in the absence of cortisol effects, correlated pre-/postsynaptic activity has changed the LTP only marginally (5th panel). Comparison with experimental data, When glucocorticoids were combined with MR antagonists, high-frequency stimulation enhanced LTP by a factor of 1.36 only (Maggio and Segal, 2007).

Our simulations show that correlated pre/postsynaptic activity leads to increased LTP at low cortisol concentrations (Fig. 4*A*). In contrast, correlated pre/postsynaptic activity leads to suppressed LTP in the presence of high cortisol concentrations (Fig. 4*B*). These results are in agreement with experiments comparing LTP in stressed and nonstressed animals using high-frequency stimulation, which is regarded to induce correlated pre/postsynaptic activity (Maggio and Segal, 2007). Our simulations also show that correlated pre/postsynaptic activity does not lead to LTP when glucocorticoids are absent (Fig. 4*C*). These results are in agreement with experimental evidence showing that the LTP induced by high-frequency stimulation was blunted when MR antagonists were added to high doses of glucocorticoids (Maggio and Segal, 2007). Thus, in case of correlated pre/postsynaptic activity, LTP shows a bell-shaped dependency on glucocorticoids: enhanced LTP occurs at low (normal) glucocorticoid concentrations, while high and very low glucocorticoid concentrations lead to suppressed LTP. The bell-shaped dependency of LTP probability on glucocorticoids has been often reported (Diamond et al., 1992; Pavlides et al., 1994, 1996; Joëls, 2006).

**Figure 5.**
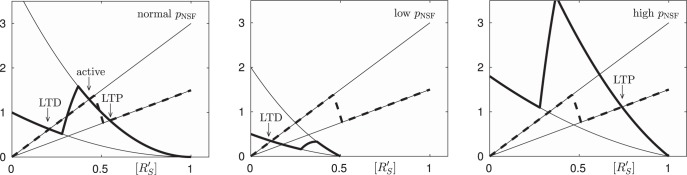
Simulation of noncorrelated pre-/postsynaptic activity at three levels of cortisol concentrations. ***A***, Low cortisol. With noncorrelated activity, proBDNF concentrations rise (4th panel, green curve). At low cortisol concentrations, the number of p75 receptors is low. When high proBDNF concentrations meet a low number of p75 receptors, p75 signaling is only modestly enhanced (3rd panel, blue curve). Consequently, protein production (R_s_ and NSF) decreases (3rd panel, red curve). As a result, the probability that NSF will bind to R_s_ decreases, so that the nonactivated form R_s_ will increase (2nd panel, the red curve becomes even higher than the green curve). Thus, these simulations show that low cortisol concentrations combined with noncorrelated pre-/postsynaptic activity produce a small LTD only (5th panel; decreased excitatory postsynaptic potentials at hour 5). Comparison with experimental data, In nonstressed animals, low-frequency stimulation evoked LTD amounting to 85% of baseline level line (Maggio and Segal, 2009). ***B***, High cortisol. With noncorrelated activity, proBDNF concentrations rise (4th panel, green curve). At high cortisol concentrations, the number of p75 receptors is high too. When high proBDNF concentrations meet a high number of p75 receptors, p75 signaling is strongly enhanced (3rd panel, blue curve). Consequently, protein production (R_s_ and NSF) is markedly decreased (3rd panel, red curve). As a result, the probability that NSF will bind to R_s_ also markedly decreases, so that the nonactivated form R_s_ will become excessively high (2nd panel, the red curve significantly exceeds the green curve). Thus, these simulations show that high cortisol concentrations combined with noncorrelated pre-/postsynaptic activity produce a marked LTD, which is less than half the baseline level (5th panel; compare panel *A*). Comparison with experimental data, In stressed animals, low-frequency stimulation produced an LTD amounting to only 49% of the baseline level line (Maggio and Segal, 2009). ***C***, No cortisol. With noncorrelated activity, proBDNF concentrations rise (4th panel, green curve). Without cortisol, the number of p75 receptors is very low, and therefore, p75 signaling is almost absent (3rd panel; black curve, which is covered by the red curve). Consequently, protein production (R_s_ and NSF) is unaltered (3rd panel, red curve). Thus, these simulations show that in the absence of cortisol effects, noncorrelated pre-/postsynaptic activity has produced a small LTD only (5th panel). Comparison with experimental data, When nonstressed animals were treated with the combination of MR and GR antagonists, low-frequency stimulation produced a LTD which was only 83% of the baseline (Maggio and Segal, 2009).

Furthermore, our simulations show that noncorrelated pre/postsynaptic activity leads to LTD at low cortisol concentrations (Fig. 5*A*). In contrast, at high cortisol concentrations, noncorrelated pre/postsynaptic activity enhanced LTD (Fig. 5*B*). These results are in agreement with the experimental evidence comparing LTD in stressed and nonstressed animals using low-frequency stimulation, which is regarded to induce only weakly correlated activity between the pre- and the postsynaptic neuron (Maggio and Segal, 2009). Our simulations also show that noncorrelated pre/postsynaptic activity does not lead to LTD in the absence of glucocorticoids (Fig. 5*C*). These results are in agreement with experimental evidence showing that noncorrelated pre/postsynaptic activity does not lead to LTD in the presence of MR and GR antagonists (Maggio and Segal, 2009). Thus, in the case of noncorrelated pre/postsynaptic activity LTD is most pronounced in the presence of high glucocorticoid concentrations, while LTD is suppressed at low (normal) or very low glucocorticoid concentrations.

**Figure 6.**
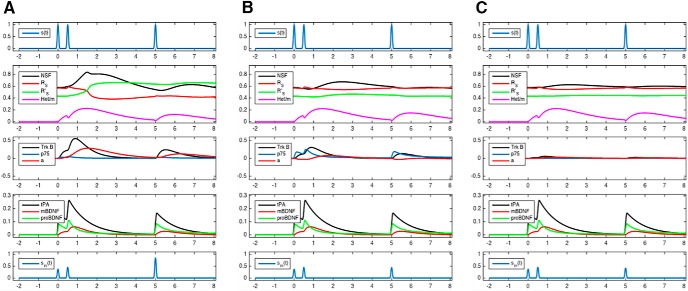
Simulations of correlated pre-/postsynaptic activity at three levels of cortisol concentrations when the insertion of long-tailed AMPA receptor subunits into the membrane is prevented. Low, high, and very low cortisol concentrations are shown in the left, middle, and right panels, respectively. The **s**imulations depicted here do only marginally deviate from the simulations obtained when the insertion of long-tailed AMPA receptor subunits into the membrane is not prevented (compare Fig. 4*A–C*).

**Figure 7.**
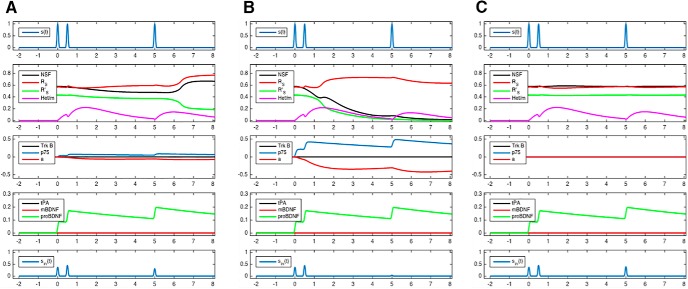
Simulations of noncorrelated pre-/postsynaptic activity at three levels of cortisol concentrations when the insertion of long-tailed AMPA receptor subunits into the membrane is prevented. Low, high, and very low cortisol concentrations are shown in the left, middle, and right panels, respectively. The **s**imulations depicted here do only marginally deviate from the simulations obtained when the insertion of long-tailed AMPA receptor subunits into the membrane is not prevented (compare Fig. 5*A–C*).

We performed additional simulations to analyze whether the insertion of AMPA receptor heteromers into the postsynaptic density is indispensable for the development of LTP and LTD. In these simulations, the model parameter *k11*, which describes the catalytic reaction for the transfer of R_S_R_L_ heteromers into the membrane, was set to zero. This means that in the simulated process the transfer of AMPA receptor heteromers into the membrane is prevented. These simulations (Figs. 6, 7) show only marginal deviations from the results obtained before (Figs. 4, 5). Thus, our previously published core model consisted of a redundant model component, i.e., the transfer of AMPA receptor heteromers into the membrane. These results are in agreement with experimental evidence showing that the transfer of AMPA receptor heteromers into the membrane is dispensable for inducing and maintain LTP (Granger et al., 2013).

## Discussion

Our modeling of synaptic plasticity shows that LTP occurs, if there is correlated activity between the pre- and postsynaptic neuron *and* glucocorticoids are in an optimal range (low normal concentrations). Optimal glucocorticoid concentrations maximize the number of TrkB receptors expressed on the postsynaptic density and correlated pre/postsynaptic activity maximizes the concentrations of mBDNF in the synaptic cleft. This association of the ligand BDNF and its TrkB receptors specifies a logical AND operation. In all other cases, when glucocorticoid concentrations are not in the optimal range or pre-/postsynaptic activity is only weakly correlated, LTP is attenuated or even LTD occurs. The results of our simulations are in agreement with the experimental evidence (Maggio and Segal, 2007, 2009).

Many biophysical models of synaptic plasticity exist, but, to the best of our knowledge, none of them has dealt with the question how glucocorticoids modulate the occurrence of LTP or LTD. The earlier of these models focused on the early phase of LTP and LTD (which is independent of protein synthesis). They were based on the assumption that LTP depends on calcium influx through NMDA-Rs (Gamble and Koch, 1987; Holmes and Levy, 1990; Zador et al., 1990). John Lisman was the one who constructed the first influential model of signal transduction pathways associated with synaptic plasticity (Lisman, 1989). His crucial question was how the same molecule, calcium, can trigger both LTP and LTD. Lisman proposed that moderate calcium levels primarily activate phosphatases that dephosphorylate Ca^++^/CaMKII, while high calcium levels promote the phosphorylation of CaMKII, and thus explain bidirectional synaptic plasticity. This hypothesis has subsequently received significant experimental support. The model of Lisman and the subsequent models of Castellani displayed a limited number of components of the signal transduction path (Lisman, 1989; Castellani et al., 2001, 2005). Bhalla and co-workers extended these basic models by including additional components (Ajay and Bhalla, 2004). Other models of the induction of synaptic plasticity were either explicitly or implicitly based on the original hypothesis of Lisman (Karmarkar and Buonomano, 2002; Shouval et al., 2002). All of these former models focused on joint pre- and postsynaptic activity, but neglected the potential role of neuromodulators.

More recent models focused on the late phase of LTP and LTD, which is dependent on protein synthesis. Some of these models have included the role of neuromodulators such as acetylcholine, noradrenaline, serotonin and dopamine (Frémaux and Gerstner, 2016), but not glucocorticoids. In the current work, we refer to data that show how glucocorticoids affect the protein synthesis in the late phase of LTP and LTD. Although the key mechanisms of the early and late phase of synaptic plasticity are different, there are structural similarities in the interaction of these mechanisms. These formal similarities become evident when comparing the model of Lisman (as described above) with ours. In our model, moderate calcium levels favor the presence of proBDNF in the synaptic cleft, thereby reducing postsynaptic protein production, while high calcium levels favor the presence of mBDNF, thereby increasing postsynaptic protein production, thus accounting for bidirectional synaptic plasticity. In view of these formal similarities between the model of Lisman and our model, it is not surprising that the predictions of both models were similar. These formal similarities suggest that the basic regulatory principle that Lisman has discovered in the early phase of LTP and LTD is also detectable in the late phase. Focusing on the mechanisms of synaptic plasticity that regulate protein production put us in a position to model the neuromodulatory effects of glucocorticoids.

The model presented here may allow for a deeper understanding of the question when and how LTP or LTD occur during stress. According to a novel framework about “uncertainty and stress,” the brain consolidates an ‘internal model of the world’ only in the absence of “expected surprise,” i.e. uncertainty (Peters et al., 2017). According to this concept, the brain minimizes its prediction errors, i.e., the differences between an organism’s predictions about its sensory inputs (embodied in its internal model of the world) and the sensations it actually encounters (Friston, 2010). The absence of uncertainty, which leads to low-normal glucocorticoid concentrations, indicates that the current internal model of the world makes accurate predictions, and thus it would make sense to consolidate this model through memory formation. Underlying such a consolidation is, among other things, e.g., dendritic spine remodeling, dendritic arbor shaping (Liston et al., 2013; Ikeda et al., 2015), the process of LTP (Joëls, 2006). In contrast, if the internal model of the world is not suitable for making accurate predictions, uncertainty will increase, as will glucocorticoid concentrations. In this case, the current internal model of the world is deconstructed and the underlying mechanisms include, among other things, e.g., dendritic arbor shrinkage (McEwen and Morrison, 2013), the process of LTD. Thus, consolidation of the internal model of the world is promoted at optimal (low normal) glucocorticoid concentrations, which indicate the absence of uncertainty or stress; in contrast, the reformation of the internal model of the world is promoted at excess glucocorticoid concentrations, which favor LTD (Peters et al., 2017). In all, our mathematical model of synaptic plasticity may provide a mechanistic explanation how the brain makes appropriate updates of its model of the world.

Our simulation study may allow predictions for future research. Learning (i.e., consolidation of memory) occurs, if the brain’s prediction errors are minimized (Friston, 2005). Accordingly, our model of synaptic plasticity predicts that “prediction-error minimization” manifests itself through maximization of the number of mBDNF molecules in the synaptic cleft, maximization of the number of TrkB receptors expressed at the postsynaptic density, maximization of postsynaptic TrkB signaling, maximization of the number of activated short-tailed AMPA receptor subunits within the cytosolic pool, and finally through maximization of LTP probability. If we consider memory consolidation as such an “optimization problem,” we will be able to design future experiments that could test the following predictions of our model: experimental interventions that systematically alter the correlation between pre- and postsynaptic activity and glucocorticoid concentrations will lead to a maximum LTP probability if and only if the synaptic mBDNF concentrations and TrkB receptors expressed at the postsynaptic density are maximal. Similarly, such interventions will lead to a maximum LTD probability, if the synaptic proBDNF concentrations and the density of the postsynaptically expressed p75 receptors are maximal.

The model presented here had already made a prediction that has been confirmed experimentally four years ago (Granger et al., 2013). Almost a decade ago, we have published the core model, which described AMPA receptor trafficking (Langemann et al., 2008). That core model was based on experimental evidence showing that regulated addition of GluA1-GluA2 and continuous replacement of GluA2-GluA3 containing synaptic AMPA receptors provide the mechanism of how surface receptor number is established and maintained (Shi et al., 2001). This experimental work was the basis of a widely-accepted view of how LTP is induced and maintained. In 2013, however, this view was challenged by experiments showing no requirement for the GluA1 C-tail for LTP (Granger et al., 2013). The GluA1 C-tail plays a key role in driving AMPA receptor heteromers into the synaptic density (Henley and Wilkinson, 2016). Because we had included these kinds of reactions in our core model (Langemann et al., 2008), we now had the opportunity for *post hoc* testing as to whether this model component was dispensable for predicting experimental evidences. To address this question, we deleted the model component that described the movement of AMPA receptor heteromers into the synaptic density as well as their removal from the synaptic density. In the current paper, we could show that after removal of this model component, our simulation results were still in agreement with the experimental evidence (Figs. 6, 7). Although our core model was based on older experimental evidence, it still was capable of predicting novel evidence that had appeared later (Granger et al., 2013).

The model presented here confines itself to describing LTP and LTD occurring at the postsynaptic sites of glutamatergic synapses. Of course, there are multiple phenomenological and mechanistic forms of LTP and LTD, which occur pre- and postsynaptically at glutamatergic and GABAergic synapses (Castillo, 2012; Yang and Calakos, 2013). At GABAergic synapses, postsynaptic GABA_B_ receptors in cooperation with VDCCs act as coincidence detectors (Gaiarsa et al., 2002; Kuczewski et al., 2010, 2011). GABA released from the presynaptic terminal activates postsynaptic GABA_B_ receptors, and back-propagating APs activate the VDCCs. On activation of the GABA_B_-VDCC complex, BDNF concentrations increase in the synaptic cleft. BDNF can also activate TrkB receptors located on presynaptic glutamatergic and GABAergic terminals (Tyler and Pozzo-Miller, 2001; Gubellini et al., 2005; Inagaki et al., 2008). At several glutamatergic and GABAergic synapses, neuronal activity can trigger enduring increases or decreases in neurotransmitter release, thereby producing LTP or LTD of synaptic strength, respectively. According to the same basic principles as presented in the current work, a future modeling of the “influence of stress on synaptic plasticity” is conceivable: It extends the present model by implementing the BDNF/TrkB system at presynaptic terminals and GABAergic synapses.

VDCCs play a role in other forms of LTP and LTD. VDCC-dependent LTP is enhanced by the same dosage of corticosterone that impairs NMDA-R-dependent LTP (Krugers et al., 2005). This species of LTP is found in the amygdala where it is believed to underlie the formation of fear memories (Blair et al., 2001). In emotional memory formation, membrane-bound MR play a crucial role in the induction of LTP (Maggio and Segal, 2012). VDCCs also constitute key mechanisms in associative LTD in the hippocampus, a form of spike time-dependent synaptic plasticity that is induced by the asynchronous pairing of postsynaptic action potentials and EPSPs (Niehusmann et al., 2010). This process is regulated by free calcium at the level of PKC and PICK1, both of which are calcium dependent. The sources of the relatively low calcium increase in LTD are VDCCs and intracellular calcium stores. Action potentials open VDCCs. Hence, in a situation where an action potential precedes the EPSP, calcium will be present at the time that glutamate activates postsynaptically the metabotropic glutamate receptor-dependent pathway (Niehusmann et al., 2010). The role of BDNF in all of these forms of NMDA-independent forms of LTP and LTD is less certain. The findings of Aarse et al. (2016) suggest that in behaving animal, the contribution of BDNF to information encoding in the form of synaptic plasticity is graded and highly dependent on experience-related factors such as the stimulus pattern and the history of synaptic experience. Nonetheless, the model presented in the current paper is limited to NMDA-dependent plasticity.

**Figure 8.**
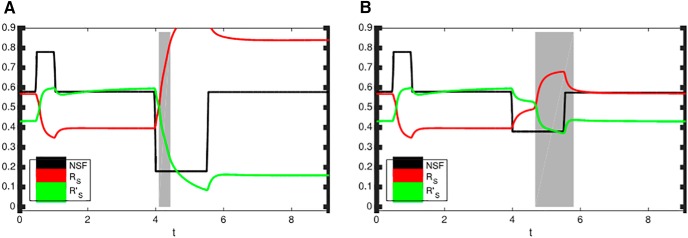
Model predictions on the likelihood of an LTP-to-LTD conversion. The simulations were performed to make predictions that could be tested in future experiments. An initial interventional increase in NSF production generates LTP (*t* < 1). Left, A strong subsequent interventional decrease of NSF is likely to convert LTP into LTD. There is an underproportionally short time interval (gray bar) not leading to LTD. Right, A small subsequent interventional decrease in NSF increases the likelihood that the synapse will not transition into LTD but into the middle active state. There is an overproportionally long time interval (gray bar) not leading to LTD.

Model-derived hypotheses should be testable by future experiments. Experimental interventions that would alter the NSF production could provide further evidence to support the proposed model with three stable stationary points representing LTP, LTD and the middle active state. A high production of NSF is predicted to drive synapses in LTP. After a subsequent reduction in NSF production (which NSF returns to the previous level), LTP is predicted to persist, and an increased amount of AMPA homomers is expected to be found in the postsynaptic density. Figure 8 shows such an initial interventional increase of NSF and the resulting changes in synapse, i.e., the transition from the middle active state to LTP (time interval before *t* = 1). In case of a subsequent interventional reduction of NSF (at time *t* = 4), the qualitative conceptual framework of the present study predicts two possible scenarios: Depending on the intensity of the subsequent NSF reduction, either LTD or the middle active state is obtained (Fig. 8*A*,*B*). A comparison may illustrate the situation: The system with three stable stationary points acts like a “skill game,” in which a small ball has to be placed in different dimples. The middle active state has a small area of attraction, and it is expected that an external influence that results in a slow change makes it possible to find the middle equilibrium in the active state, but not a rapid change.

Figure 8*A* shows a strong subsequent experimental reduction of NSF, and the system occupies the stationary stable point related to LTD, as discussed above. The simulation shows that a rather short time interval of a strong P_NSF_ reduction is sufficient to transfer the synaptic system into LTD. The gray bar indicates the length of the time interval during which the system does not move to LTD. The simulations show that there are only a few interval lengths that are not associated with LTD. These simulation results agree with the interpretation that a strong reduction of NSF leads to a rapid system change. Consequently, it is predicted that under such conditions the system will occupy the LTD equilibrium in most cases; it appears rather rare and only possible for short P_NSF_ reductions that the system will not move into LTD.

In contrast, Figure 8*B* shows a comparatively small subsequent experimental reduction of NSF. The simulations show that the system has an overproportionately large range of time interval lengths (marked by the gray bar) that is not associated with the LTD equilibrium. It is not fully clear whether there are additional mechanisms, which are not modeled here, preventing the system from falling back into the active state. But according to our model, a modest subsequent interventional reduction of NSF is less likely to cause LTD.

In all, the model-derived hypothesis that duration and intensity of a particular experimental intervention determine which of the three equilibrium states in the synaptic plasticity is occupied can be directly tested in future experiments. Such an approach could test the internals of the model presented here.

Reducing model complexity, while maintaining model accuracy, is always an important issue (Friston, 2010). As shown above, novel evidence helps to remove dispensable elements from a model without affecting the model’s ability to make accurate predictions. According to Occam’s razor the model is best when it makes accurate predictions with minimal complexity. Of course, we are aware that the process of synaptic plasticity involves a large number of different molecules. However, the question is whether all these mechanisms are necessary for predicting experimental results. With the model presented here we tried to keep the number of factors that affect LTP or LTD at a minimum. The complexity of the model presented here is relatively low, but the model components are sufficient to accurately predict the experimental results (Maggio and Segal, 2007, 2009).

In conclusion, our simulations may help to explain how glucocorticoids and pre/postsynaptic activity govern the occurrence of NMDA-R-dependent LTP or LTD. The joint mechanism in that process appears to be the interaction between BDNF and BDNF receptors. Full LTP occurs if glucocorticoids are in an optimal (low normal) range and activity between the pre- and postsynaptic neuron is highly correlated. During stress, however, high glucocorticoid levels suspend these learning processes. Our findings may provide a mechanistic basis for understanding how the brain controls learning under uncertainty.

## Synthesis

Reviewing Editor: Cheryl Sisk, Michigan State University Decisions are customarily a result of the Reviewing Editor and the peer reviewers coming together and discussing their recommendations until a consensus is reached. When revisions are invited, a fact-based synthesis statement explaining their decision and outlining what is needed to prepare a revision will be listed below. The following reviewer(s) agreed to reveal their identity: Claus Normann.

Two experts have reviewed your manuscript and have made recommendations for a revised manuscript. I have synthesized the reviewers' comments and recommendations below. In general, both reviewers raised questions about the generalizability of the model to LTP/LTD more broadly, and they thought that the conclusions drawn about the utility of the model were over-reaching (item 1). Other concerns were around the predictive value and experimental testability of the model (items 2-3). Items 4-5 include other suggestions for revising the discussion. I note that the JN editors' comments reflected similar concerns, and even though you made changes to your manuscript to address them, these issues remain problematic. If you believe you can satisfactorily address the reviewers' concerns, please revise your manuscript as appropriate and I will ask for a re-review. If you resubmit this manuscript, please provide the code used to generate your results (item 6).

1. The authors have extended their earlier (2008) mathematical model of plasticity by including modulation by cortisol. It is certainly of interest to understand how synaptic plasticity may be modulated by other factors. Nevertheless, the significance of this work is over-stated. There remains considerable controversy over how well LTP, as induced mostly in brain slice culture using unnatural stereotyped paired stimuli, represents the actual complex processes of memory formation, which also involves hippocampal replay and hippocampal-cortical communication. However, LTP in tissue slices is a focus of much research, and therefore models are worth exploring.

The experience of ‘stress’ seems to involve other neuromodulators, such as ACh, dopamine and norepinephrine, which are widely believed relevant to synaptic plasticity. The levels of other neuromodulators seem likely to rise in some of the same situations as those that elevate cortisol. This article makes no mention of any of these modulators; very likely such a complex model would become analytically intractable. Nevertheless, it seems that the biological relevance of the tractable simpler model presented in this MS to real brain function is in doubt. Their work may be better described as one model probably relevant to LTP in tissue culture, and possibly relevant to memory. In the discussion, the authors oversimplify the role of LTP and LTD for memory formation in a way that LTP consolidates memory. Rather, both LTP and LTD and their interaction might build information consolidation.

The authors describe a model of synaptic plasticity that can be used to predict if LTP or LTD is induced. It depends on pre- and postsynaptic spike timing and stress, which is expressed by glucocorticoid levels, which in turn affects BDNF signaling. In my opinion, this model is best suited to explain the stress modulation of NMDA-dependent synaptic plasticity. However, spike time-dependent plasticity is not uniformly NMDA dependent. In a situation, where pre- and postsynaptic activity is asynchronous, an opening of NMDA receptors is not expected and the Ca2+ influx into the postsynaptic cell is carried via HVA Ca2+ channels, using different coincidence receptors (PKC, PICK1; e.g. in Niehusmann et al, 2010). The BDNF-dependence of these forms of NMDA-independent forms of plasticity is much less clear and the facilitation of LTD by stress might dependent on the modulation of Ca2+ channel conductivity (compare the work of Joels and coworkers). Thus, the authors should focus their arguments on NMDA-dependent plasticity and not on spike-time dependent forms of plasticity and should at least discuss other mechanisms of LTD induction which are not incorporated in their model. Even the Woo paper describes a role for p75 only for NMDAR dependent forms of LTD. Aarse et al 2016 describe that BDNF is not required for all forms of hippocampal plasticity, please discuss. A more clinical argument against a universal role of BDNF in plasticity comes from the mixed evidence for a role of the ValMet polymorphism in depression which can be conceptualized as a stress disorder where a pathogenetic role of synaptic plasticity has been repeatedly demonstrated (work of Castren's group).

The authors should generally down-tune the language of their manuscript and avoid phrases as “As early as in 2008, we have published...”, “without any miraculous memory tag...” or “our model has already made an important prediction”.

2. LTP/LTD is more complicated than Hebb's Rule: even 16 years ago Bi and Poo documented many empirical large departures from the Hebb rule in their 2001 article on LTP/LTD: “Synaptic modification of correlated activity: Hebb's postulate revisited” (Ann. Rev. Neurosci., 24:139-166). There are several other models of synaptic plasticity mechanisms. The authors make little mention of them. Does their model predict different phenomena than other models? At this point, after years of development, the authors should situate their model in the landscape of other models for the phenomenon.

3. It is not obvious how experimenters could probe or test the internals of the model presented here. What experiments could be done to estimate one of the parameters in their model? Is there some experiment that would expose the inner workings of their model? Is there some experiment that the model would predict would give a substantially different result from ‘common sense’? Does this model yield any novel insight or non-obvious testable predictions? The main conclusion of the paper seems aptly summarized in lines 389-390: “Full LTP occurs if glucocorticoids are in an optimal (low normal) range and activity between the pre- and postsynaptic neuron is highly correlated. In all other cases learning is suspended. ”The authors say very similar things at several points in the paper. However this is more or less what we would expect based on experience with most physiological systems: modest levels of stress enhance function, while extreme levels impair function, in the well-known ‘inverted U’. So, this obvious prediction can't separate their model from other models, or from qualitative reasoning.

4. Some comparisons with experimental outcomes are noted, mostly with studies by Maggio and Segal done 8 and 10 years ago) but these comparisons are qualitative, (a measure increases or decreases) rather than even roughly quantitative (e.g. predicting a two-fold vs. five-fold increase). The majority of the interest seems to be in the equations rather than in the biological meaning. Nevertheless the equations are not analyzed in algebraic terms as with simpler dynamic systems; rather results are presented by simulation. That is probably due to complexity; is it? The discussion of stabile points (which imply a bifurcation of plasticity dynamics; lines 234-250) seem promising but are under-developed. What consequences for LTP or normal plasticity would these have? Could experimenters push these bifurcations in some non-obvious way?

5. It is not clear to this reviewer that the discussion in lines 345-355 of predictions of the older ‘core’ model belong in this paper, although perhaps interesting. In my opinion, the reference to “Friston's 'free energy principle'” in the discussion is superfluous, because very few readers of this journal will be familiar with this idea or in a position to interpret the claim.

6. While the figures are clear and apt, it is becoming increasingly common for quantitative and simulation studies to include the code the authors used to generate the main results, so that others can reproduce and extend the results with a minimum of trial-and-error.

## Author Response

Dear Prof. Sisk,

Thank you for giving us the opportunity to resubmit our manuscript 'LTP or LTD? Modelling the influence of stress on synaptic plasticity' to eNeuro (eN-TTNC-0242-17X). We appreciate the valuable comments and questions that you and the reviewer have asked. We have revised the manuscript in the following manner:

1. Both reviewers raised the questions about the generalizability of the model to LTP/LTD and thought that the conclusions drawn about the utility of the model were over-reaching.

With respect to this critic we revised the abstract, the significance statement, the introduction and the discussion: We clearly stated that we 'focus on N-methyl-D-aspartate receptor (NMDA-R) dependent LTP and LTD, two forms of activity-dependent long-term changes in synaptic efficacy that have been extensively studied in brain slice cultures'.

We inserted a new paragraph in the introduction (lines 78 - 92) where we included 'spike-timing dependent plasticity' and also mentioned the role of voltage dependent calcium channels (VDCC). In addition, we included a new paragraph in the discussion (lines 442 - 458) focusing on VDCCs and the potential role of BDNF (also referring to the work of Aarse). In this way, we discussed mechanisms operate in other forms of LTP and LTD, always emphasizing that our model is limited to NMDA-R dependent synaptic plasticity.

Finally, we down-tuned the language in the manuscript.

2. The reviewers criticized that LTP/LTD is more complicated than Hebb's rule.

We agree and thus we replaced the former paragraph in the introduction by a new paragraph (lines 78 - 92), which also refers to other forms of synaptic plasticity mechanisms.

It was also criticized that we did not place our model in the landscape of other models for the phenomenon.

Therefore, we inserted two new paragraphs in the discussion (lines 346 - 377), which give an overview over the biophysical models of synaptic plasticity that exists so far. In addition, we related our findings to the predictions of these existing models.

3. Furthermore, it was criticized that it is not obvious how experimenters could probe or test the internals of the model presented.

To address this issue, we included new paragraphs in the discussion (lines 459 - 492). In that part, we presented simulation results that were specifically obtained due to your critic; these simulations generated predictions for future model testing. The simulation results are shown in a new figure (Figure 7A and B). As outlined in this part, the model-derived hypotheses can be directly tested in future experiments.

4. It was criticized that the comparison between our model predictions and the experimental results of Maggio and Segal were 'qualitative' only, instead of being 'roughly quantitative'.

To address this issue, we revised the figure legends of figure 2 and figure 3. Here we showed 'roughly quantitative comparisons' between our simulations and the experimental results: "For example (lines 583 - 586): "full-magnitude LTP of approximately double size as compared to baseline. Comparison with experimental data: In non-stressed animals, high frequency stimulation enhanced LTP by a factor of 1.69 compared to baseline (Maggio and Segal, 2007)."

It was furthermore proposed to extend the discussion of stable points (which imply a bifurcation of plasticity dynamics).

To address this issue, a new paragraph has been included into the method section within the chapter 'model properties' (lines 246 - 271). This new paragraph gives a more detailed mathematical analysis when and how the system moves into its stable stationary points.

It was asked whether experimenters could push these bifurcations in some non-obvious way.

To address this problem, we have proposed new experiments that change the production rate of the NSF. We predicted - according to our model - when and how the system would move from one stable stationary point into another one. Details of these predictions are outlined in the discussion (lines 459 - 492) and Figure 7A and B.

5. It was questioned whether the predictions of the older core model belong in this paper.

We believe that the findings of Granger et al. (2013) have challenged a long-held view on AMPA receptor trafficking. These findings have prompted a debate in the scientific community. We think that the ability of our core model (2008) to predict the findings of Granger supports its validity. Therefore, we propose to keep this short issue in the discussion.

It was criticized that Karl Friston's 'free energy principle' in the discussion is superfluous.

To address this issue, we removed the term 'free energy' from the manuscript. Instead, we focused on the conception that memory formation can be regarded as an 'optimization problem' (lines 403 - 409). Against this background, we proposed experiments that could directly test whether a system that shows an optimization of BDNF concentrations in the synaptic cleft and an optimization of the number of TrkB receptors in the postsynaptic density also exhibits optimized conditions for LTP. In addition to the experimental designs described in the discussion (lines 459 - 492), these ideas provide further opportunities to test the internals of the models presented here.

6. We included the code so that other researchers can reproduce and extend the results. We inserted the code accessibility statement in the method section. We also included a legend for the code uploaded as Extended Data in the manuscript with the figure legends.

We hope we could answer all the questions and comments. We are looking forward to learning your final decision.

Yours sincerely,

Achim Peters
